# Changes in Adverse Pregnancy Outcomes Associated With the COVID-19 Pandemic in the United States

**DOI:** 10.1001/jamanetworkopen.2021.29560

**Published:** 2021-10-15

**Authors:** Shengzhi Sun, David A. Savitz, Gregory A. Wellenius

**Affiliations:** 1 Department of Environmental Health, Boston University School of Public Health, Boston, Massachusetts; 2 OptumLabs Visiting Scholar, OptumLabs, Eden Prairie, Minnesota; 3 Department of Epidemiology, Brown University School of Public Health, Providence, Rhode Island

## Abstract

This cross-sectional study evaluates the change in rates of pregnancy complications during the COVID-19 pandemic among pregnant women with commercial health insurance across the US.

## Introduction

The COVID-19 pandemic and ensuing government response has led to profound changes in lifestyle, physical and mental health, and health care access and delivery. The cumulative impact of these stressors on the risk of adverse pregnancy outcomes has not been examined in detail, and initial evidence has been inconsistent.^1,2,3,4^ Accordingly, we evaluated the change in rates of pregnancy complications associated with the pandemic period among pregnant women with commercial health insurance across the US.

## Methods

This cross-sectional study involved analysis of deidentified data and was deemed nonhuman participants research by the institutional review board at Boston University, and, therefore, informed consent was waived. This analysis followed the Strengthening the Reporting of Observational Studies in Epidemiology (STROBE) reporting guideline.

We identified medical claims indicative of a delivery (ie, *International Statistical Classification of Diseases, Tenth Revision *[*ICD-10*] Z37.xx) and adverse pregnancy outcomes, which have been previously defined (eAppendix in the Supplement).^5^ We analyzed claims from January 1, 2019, to December 31, 2020, from the OptumLabs Data Warehouse, a longitudinal database of deidentified administrative claims among commercial and Medicare Advantage enrollees throughout the US. Claims separated by 182 days or more were considered separate pregnancies.

We defined the COVID-19 pandemic period as March 1 to December 31, 2020, and compared the proportion of deliveries with each adverse outcome during this period vs the analogous period in 2019 (ie, referent period). We obtained risk ratios (RR) and 95% CIs using Poisson regression with the logarithm of number of deliveries as an offset. We performed sensitivity analyses restricted to women who were continuously enrolled in a commercial health insurance plan from March 1, 2019, to December 31, 2020. We also examined geographic variation in the RR by fitting a separate model in each state and using a random-effects meta-analytic model to examine statistical heterogeneity across states. We conducted analyses in R version 3.6.1 (R Project for Statistical Computing) and considered a 2-sided *P* < .05 as statistically significant. Data analyses took place from May to August 2021.

## Results

We identified 152 903 deliveries during the COVID-19 pandemic period and 172 095 deliveries during the referent period. The most common documented adverse outcome was premature rupture of membranes (33 392 of 324 998 deliveries [10.3%]) followed by gestational diabetes (30 255 of 324 998 deliveries [9.3%] ) and gestational hypertension (27 602 of 324 998 deliveries [8.5%]) (Figure).

**Figure.**
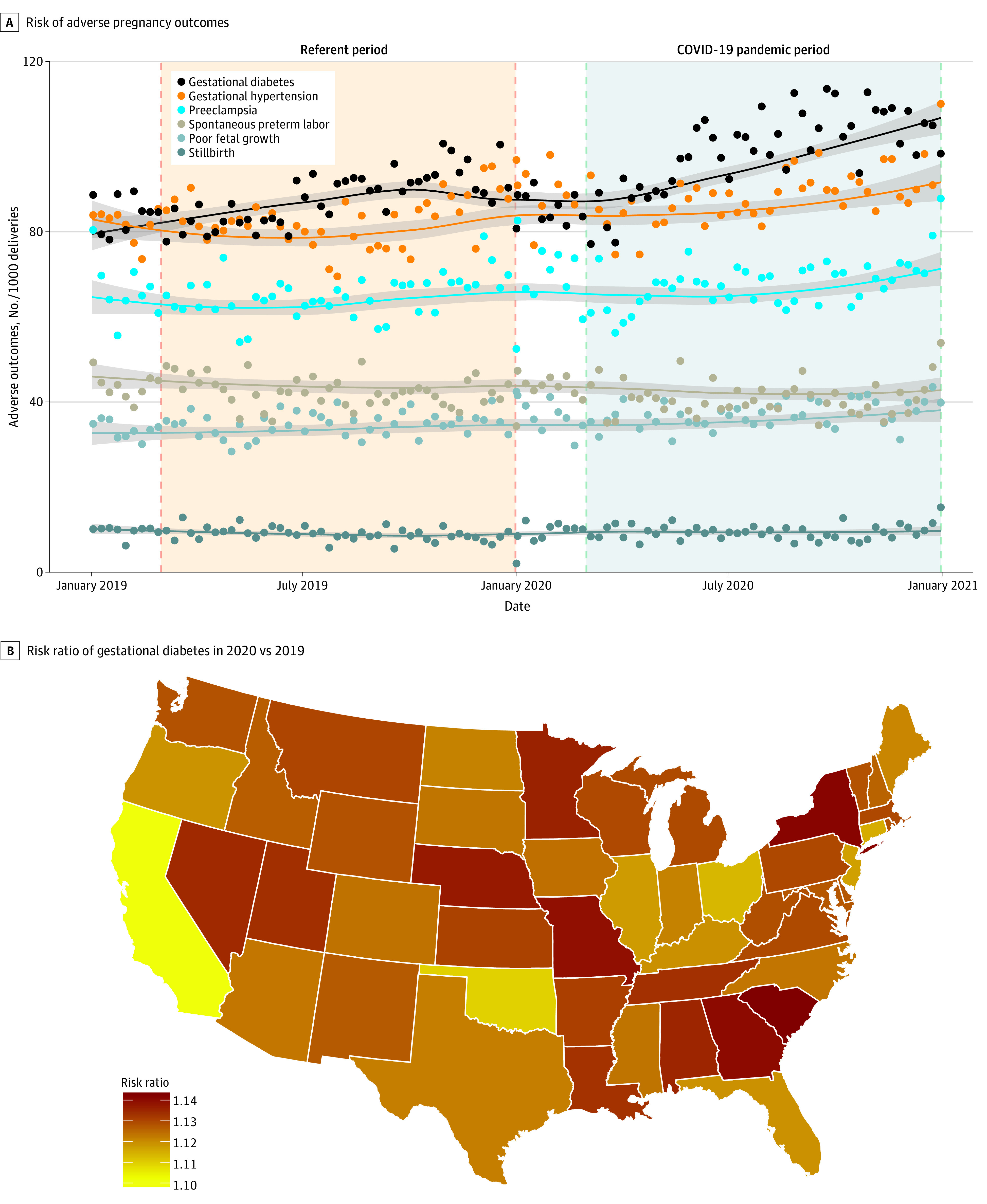
Risk of Adverse Pregnancy Outcomes and Risk Ratio of Gestational Diabetes in the United States During the COVID-19 Pandemic Panel A shows the risk of adverse pregnancy outcomes in the United States from January 1, 2019, to December 31, 2020. The circles indicate weekly mean number of adverse pregnancy outcomes per 1000 deliveries. Solid lines show the time trend by fitting the Loess smooth function based on daily number of adverse pregnancy outcomes. Panel B compares the risk ratio of gestational diabetes during the pandemic period (ie, March 1, 2020, to December 31, 2020) vs the referent period (March 1, 2019, to December 31, 2019).

Compared with the referent period, the pandemic period was associated with a statistically significant higher risk of gestational diabetes (RR, 1.12; 95% CI, 1.10-1.15), gestational hypertension (RR, 1.07; 95% CI, 1.05-1.09), poor fetal growth (RR; 1.07; 95% CI, 1.03-1.11), and preeclampsia (RR, 1.04; 95% CI, 1.01-1.07) (Table). Results were similar in sensitivity analyses restricted to 186 464 women (57.4%) who were continuously enrolled in a commercial health insurance plan throughout the study period. We found little evidence of heterogeneity across states, except for gestational diabetes (*I*^2^ = 9.5%, *P* = .04) (Figure).

**Table.** Absolute and Relative Risk of Delivery With an Adverse Outcome During March 1 to December 31, 2020, vs the Same Period in 2019^a^

Pregnancy outcomes	All deliveries (N = 324 998)				Deliveries among women continuously enrolled in a health plan between March 2019 and December 2020 (n = 186 464)			
	No. per 1000 deliveries		Risk ratio (95% CI)	*P* value	No. per 1000 deliveries		Risk ratio (95% CI)	*P* value
	2019	2020			2019	2020		
Premature rupture of membranes	102.0	103.6	1.02 (0.99-1.04)	.14	101.1	101.7	1.01 (0.98-1.03)	.68
Placental abruption	9.2	9.6	1.04 (0.97-1.12)	.23	9.0	9.3	1.03 (0.94-1.14)	.50
Preeclampsia	64.7	67.3	1.04 (1.01-1.07)	.003	62.4	65.9	1.06 (1.02-1.09)	.002
Gestational hypertension	82.2	88.0	1.07 (1.05-1.09)	<.001	81.8	86.5	1.06 (1.03-1.09)	<.001
Gestational diabetes	88.0	98.9	1.12 (1.10-1.15)	<.001	89.0	100.5	1.13 (1.10-1.16)	<.001
Poor fetal growth	34.7	37.1	1.07 (1.03-1.11)	<.001	32.7	35.8	1.10 (1.05-1.15)	<.001
Stillbirth	8.9	9.3	1.04 (0.97-1.11)	.32	8.8	8.9	1.01 (0.92-1.11)	.88
Spontaneous preterm labor	42.4	40.6	0.96 (0.93-0.99)	.01	41.1	40.0	0.97 (0.93-1.02)	.24

^a^ The corresponding *International Statistical Classification of Diseases, Tenth Revision* diagnosis codes for adverse pregnancy outcomes can be found in eAppendix in the Supplement.

## Discussion

To our knowledge, this is the largest and most comprehensive study to date of the impact of the COVID-19 pandemic on adverse pregnancy outcomes. Our finding that the pandemic period was not associated with a changing risk of stillbirth and provided only modest evidence of a lower risk of preterm birth is broadly consistent with the existing literature.^1,3^ Our study provides novel evidence of the association of the pandemic with the risk of complications that have rarely been documented, including gestational hypertension, poor fetal growth, and preeclampsia.

This study had limitations, including the reliance on billing codes from health care claims to ascertain complications of pregnancy, the lack of data on gestational age at the time of birth, and the inability to ascertain iatrogenic preterm deliveries and COVID-19 status. Our results may not generalize to pregnant women without commercial health insurance or those outside of the US.

## Notes

Supplement. **eAppendix.** Adverse Pregnancy Outcomes and Corresponding *International Classification of Diseases, Tenth Revision* (*ICD-10*) Diagnosis
