# Supplementary material for: Changes in Adverse Pregnancy Outcomes Associated With the COVID-19 Pandemic in the United States
Source: JAMA Netw Open. 2021 Oct 15;4(10):e2129560. doi: 10.1001/jamanetworkopen.2021.29560 (PMC8520131; doi:10.1001/jamanetworkopen.2021.29560)
Supplement: Supplement. — eAppendix. Adverse Pregnancy Outcomes and Corresponding International Classification of Diseases, Tenth Revision (ICD-10) Diagnosis [file jamanetwopen-e2129560-s001.pdf]

Supplemental Online Content

Sun S, Savitz DA, Wellenius GA. Changes in adverse pregnancy outcomes associated with the COVID-19 pandemic in the United States. *JAMA Netw Open*. 2021;4(10):e2129560.
doi:10.1001/jamanetworkopen.2021.29560

eAppendix. Adverse Pregnancy Outcomes and Corresponding *International Classification of Diseases, Tenth Revision* (*ICD-10*) Diagnosis

This supplemental material has been provided by the authors to give readers additional information about their work.

© 2021 Sun S et al. *JAMA Network Open.*

eAppendix. Adverse Pregnancy Outcomes and Corresponding *International Classification of Diseases, Tenth Revision* (*ICD-10*) Diagnosis

**Adverse pregnancy outcomes**	**ICD-10 diagnosis**
Delivery outcome	Z37.xx
Premature rupture of membranes	O42.xx
Preeclampsia	O11.xx, O14.xx, O15.xx
Gestational hypertension	O13.xx, O16.xx
Gestational diabetes	O99.81, O24.4x
Poor fetal growth	O36.5x
Placental abruption	O45.xx
Stillbirth	Z37.1x, Z37.3x, Z37.4x, Z37.7x
Spontaneous preterm labor	O60.1x

© 2021 Sun S et al. *JAMA Network Open.*
